# Identification of novel lactate metabolism signatures and molecular subtypes for prognosis in hepatocellular carcinoma

**DOI:** 10.3389/fcell.2022.960277

**Published:** 2022-09-02

**Authors:** Qiutong Guan, Jing Pan, Ninghui Ren, Chu Qiao, Minjie Wei, Zhenhua Li

**Affiliations:** Department of Pharmacology, School of Pharmacy, China Medical University, Shenyang, Liaoning, China

**Keywords:** hepatocellular carcinoma, lactate metabolism, lncRNAs, molecular subtype, prognostic model

## Abstract

**Background:** Evidence has shown that lactate, an immune signaling molecule, is associated with hepatocellular carcinoma (HCC) progression and immune suppression. Therefore, identifying lactate metabolism-related molecules is a promising therapeutic strategy to inhibit the development of HCC and overcome chemotherapy resistance. Long noncoding RNAs (lncRNAs) are related to tumorigenesis and metastasis. Hence, verifying the molecular subtypes of lncRNAs related to lactate metabolism will play a critical role in managing HCC.

**Methods:** Based on HCC data in The Cancer Genome Atlas (TCGA), lactate metabolic pathway-related genes were enriched by gene collection and enrichment analysis (GSEA). Lactate metabolism-related lncRNAs (LM_lncRNAs) were identified by correlation analysis, HCC molecular subtypes were determined using nonnegative matrix factorization (NMF) clustering, and the response of the three subtypes to chemotherapeutics was further evaluated using the Genomic Tumor Sensitive Cell Line (GDSC) dataset. LM_lncRNAs were examined via Lasso-Cox regression analysis to determine prognosis for patients. A Nomagram plot was used to predict patient survival time.

**Results:** Three molecular subtypes of HCC were identified. The survival rate of patients with C1 subtype was higher than that of those with C2 and C3. Additionally, patients with C3 subtype have higher levels of immune cell infiltration and high expression of genes related to immune checkpoints. The GDSC results indicated that patients with C3 subtypes were more sensitive to chemotherapy drugs such as sorafenib and sunitinib. The prognostic risk assessment model consisted of six risk factors (AC034229.4, AC131009.1, MYOSLID, AC008667.1, AC012073.1, AC068025.1) and two protective factors (LINC00402 and AC103858.1). Based on *Kaplan-Meier* analysis, low-risk HCC patients had a high survival rate, and the receiver operating characteristic curve (ROC), calibration curve, and C-index confirmed good prediction ability.

**Conclusion:** In this study, the molecular subtyping method and prediction model of lactate metabolism-related lncRNAs (LM_lncRNAs) were constructed for the prognosis of HCC patients. This work demonstrated the potential targets of LM_lncRNAs and provided a novel perspective and therapeutic paradigm for future clinical translation.

## Introduction

Hepatocellular carcinoma (HCC) ranks in the top six malignant tumor incidences and the top three mortality rates yearly, posing a severe threat to human health. Globally, 821,700 liver cancer deaths occurred in 2020, accounting for 8.3 percent of cancer cases (Siegel et al., 2020; Sung et al., 2021). New HCC cases account for 55% of the global total in China, and approximately 422,100 patients die from HCC each year (Chen et al., 2016). The current clinical treatment of HCC mainly includes surgery, chemotherapy, and immunotherapy (Anwanwan et al., 2020). Because early-stage HCC is usually asymptomatic, only 5–15% of patients may be surgically removed, but they are prone to recurrence after surgery. Most patients in the middle and late stages have various degrees of vascular invasion. The surgical resection rate is low, and the 5-year survival rate of patients is below 20% (Maluccio and Covey, 2012). Targeted therapies such as sorafenib are selected for advanced HCC patients, but long-term use is prone to toxic side effects and drug resistance (El-Serag et al., 2008). Although immunotherapy for HCC has achieved some success, it still has limitations, such as a low objective remission rate and side effects.

As the most prominent metabolic organ in the human body, the liver plays a vital role in many physiological processes and maintaining metabolic homeostasis. The liver stores the body’s glucose, either from glycogen or lactate (muscle), glycerol (adipose tissue), amino acids (gut and muscle), etc. (Trefts et al., 2017). The Warburg effect is one of the characteristics of tumor metabolism. With sufficient oxygen, tumor cells consume glucose to provide energy for HCC cells and generate a large amount of lactate, resulting in a low-glucose metabolic environment. Studies have confirmed that an acidic tumor microenvironment (TME) is more conducive to highly aggressive tumor cell subtypes and ultimately promotes tumor development (Huang et al., 2016). It was reported that lactate might induce vascular endothelial growth factor expression and M2 polarization of tumor-associated macrophages (Colegio et al., 2014). The macrophage lactate/ATP6V0d2/HIF-2α axis is critical in human patients’ signaling and tumor growth. In addition, lactate accumulates on the membrane by activating monocarboxylate transporters (MCTs), especially MCT4, forming an acidic TME that inhibits antitumor immune responses (Halestrap, 2012). Moreover, lactate may modulate immune responses, affecting the function and survival of NK and T cells and promoting immune escape (Brand et al., 2016). These findings suggest that lactic acid promotes the proliferation and invasion of tumor cells and has an immunosuppressive effect. Thus, it is necessary to screen critical molecules related to early diagnosis, survival prediction, and lactate metabolism in the HCC TME and develop novel and effective strategies for treating primary and secondary HCC.

Recently, high-throughput sequencing technology has gradually become an essential tool in identifying clinically actionable biomarkers and regulators for predicting monitoring and clinical stratification. Several researchers have linked metabolomics to the genome, enabling studies to discover and identify metabolites (Liu et al., 2021). Many studies have developed prognosis prediction models based on a variety of biomarkers, most of which focus on hypoxia’s impact on immune responses (Kim et al., 2019; Zhang et al., 2020a).

Long noncoding RNAs (lncRNAs) are a series of transcriptional RNAs with over 200 nucleotides that have no protein-coding ability (Iyer et al., 2015). They are involved in tumorigenesis in various cancers, including HCC (Ally et al., 2017). For example, lncRNA HULC is upregulated and promotes HCC progression, metastasis, and resistance (Klec et al., 2019). A lncRNA activated by TGF-β (lncRNA-ATB) was associated with poor outcome in metastatic HCC (Yuan et al., 2014). These studies have demonstrated that lncRNAs have regulatory effects on the metabolism of HCC. Therefore, lncRNAs are considered novel drug screening targets showing promising research prospects and may be used as one of the most sensitive and specific key biomarkers to establish the prognosis of HCC. However, few studies have addressed the impact and prognostic value of lactate metabolism-related lncRNAs (LM_lncRNAs) in the progression of HCC.

In the present study, by integrating bioinformatics with potential LM_lncRNAs in HCC, three subtypes of patients with different clinical characteristics were clustered and evaluated. In this study, a prognostic model was developed to differentiate between high- and low-risk HCC patients. The results are promising and may be helpful in developing comprehensive treatment modules for HCC patients.

## Materials and methods

### Data acquisition

The Cancer Genome Atlas (TCGA) access policies and guidelines were followed. HCC expression data were obtained from the official TCGA website (https://portal.gdc.cancer.gov), including 407 HCC and 58 normal samples. We excluded data lacking survival information and data with a follow-up time of fewer than 30 days to reduce the death of patients due to other causes and finally retained a sample of 374 patients (Table 1).

**TABLE 1:** The clinical characteristics of patients in the TCGA dataset.

Variable	Number of samples
Gender	
Male/Female	246/128
Age	
≤65/>65/UN	216/127/31
Stage	
I/II/III/IV/UN	178/85/81/8/22
Grade	
G1/G2/G3/G4	53/161/112/12/36
T	
TX/T1/T2/T3/T4/UN	1/185/93/80/13
M	
M0/M1/MX	271/6/97
N	
N0/N1/NX/UN	263/7/103/1

First, the downloaded Fragments Per Kilobase Million (FPKM) data were converted into Transcripts Per Million (TPM) format, and all genes in the pathway with a *p* < 0.05 were selected. The lactate-related gene set (Hallmark-lactate) was analyzed based on the Molecular Signatures Database (MSigDB database, https://www.gsea-msigdb.org/gsea/index.jsp) (Liberzon et al., 2015). Gene Set Enrichment Analysis (GSEA) was used to determine the gene expression in the HCC and normal groups (Subramanian et al., 2005). Among the eight pathways, 4 with *p* < 0.05 were included as significantly different pathways, including 243 genes.

### Correlation analysis

Clinical information and lncRNA expression were combined for downstream analysis. Gene ontology (GO) and Kyoto Genome Encyclopedia analyses (KEGG) of lactic acid mRNAs in HCC. Using Pearson correlation, a relationship between the significantly lactate metabolism-related genes and all differentially expressed lncRNAs was calculated. Correlations were considered if |*R* ^2^| > 0.3 and *p* < 0.001. Similarly, up- and down-regulated differentially expressed lncRNAs were acquired using limma (R package), and the lncRNAs were identified as HCC LM_lncRNAs (Ritchie et al., 2015). The screening criteria for differential analysis were false discovery rate (FDR) < 0.05 and |log_2_ Fold Change| > 1 (|log_2_ FC| > 1).

### Non-negative matrix factorization (NMF) clustering

To determine the subtypes of LM_lncRNAs in HCC, we used an NMF clustering algorithm to select lncRNAs with significant differences in expression (*p* < 0.05, |log_2_FC|>1) through the “NMF” R package to cluster HCC samples (Gaujoux and Seoighe, 2010). The NMF algorithm in bioinformatics is an efficient method for reducing the dimensionality of data such as gene expression microarrays. As an efficient way of dimensionality reduction, The cluster k value is between 2 and 10, and the optimal k value is three according to the affinity coefficient. The principal component analysis (PCA) was used to verify the validity of the classification. In order to investigate the correlation between genes that are related to lactate metabolism and the TME, we further evaluated the relationship between different LM_lncRNA molecular subtypes and immune cell infiltration levels by CIBERSORT.

### Development of the lactate metabolism-related lncRNAs prognostic signature

The “survival” package investigated the correlations between LM_lncRNA scores and overall survival. The R software package limma explored differential lncRNA analysis between HCC and non-HCC tissues, and the screening conditions were *p* < 0.05 and |log_2_FC| > 1. Univariate Cox analysis, least absolute shrinkage, and selection operator (Lasso) Cox were successfully used to reduce the selected genes to establish a survival risk prediction model for HCC. We sampled the dataset with 2000 replacements and selected markers that recurred more than 1,000 times for the Lasso-penalty Cox regression selection operator. Multivariate Cox analysis was utilized to optimize using the survminer software package for feature selection, we assessed lncRNAs as independent prognostic factors for patient survival by combining them with clinical variables. A stepwise approach is used to select the optimal model further. A prognostic risk score for eight LM_lncRNAs was calculated through multiplying the multivariate Cox regression model with its linear combination of expression levels. The expression of the model is:

Risk score = @Expr of lncRNA 1) × coefficient of lncRNA (1)] + @Expr of lncRNA 2) ×coefficient of lncRNA (2)] + …… + @Expr of lncRNA (n) × coefficient of lncRNA (n)]

$$Riskscore=\sum_{i=0}^{N}\left(Expi*\beta i\right)$$ (1)

Expi and βi are each prognostic lncRNA’s expression level and coefficient, respectively.

Patients were divided into high- and low-risk groups based on the median risk score. Lactic acid-related scores were also determined, and a forest map was created. Using *Kaplan-Meier* analysis, the receiver operating characteristic curve (ROC), and the C-index, the rationality of the model under different molecular subtypes were verified.

### GSEA enrichment analysis and the predictive nomogram

By using the Kyoto Gene and Genome Encyclopedia (KEGG) pathway, GSEA was used to analyze the genes within the high/low-risk groups. Then, we searched the TCGA-HCC database for these features. The survival of patients over the next 1, 3, and 5 years was predicted using RMS packages and calibration statistics. An analysis of statistical data was conducted, as well as calibration curves to verify the accuracy of 3-year and 5-year patient predictions.

### Immunity and gene expression

The CIBERSORT (Charoentong et al., 2017), ESTIMATE (Yoshihara et al., 2013), single-sample gene collection and enrichment analysis (ssGSEA), and TIMER (Li et al., 2017) algorithms were widely used to evaluate the cellular composition and cellular immune response between each cell subtype. CIBERSORT is an analytical tool to impute gene expression profiles and estimate the abundances of member cell types in a mixed cell population using gene expression data. ESTIMATE provides researchers with scores for tumor purity, the level of stromal cells present, and the infiltration level of immune cells in tumor tissues based on expression data. With TIMER, the immune infiltrates within cancers of diverse types can be systematically analyzed. TIMER is used to estimate the abundance of six immune cells (B cells, CD4^+^ T cells, CD8^+^ T cells, Neutrophils, Macrophages, and Dendritic cells). The samples of each patient are scored based on the algorithm of these websites to calculate the correlation with immunity. Different algorithms produce different immune responses, as shown by heatmaps. Additionally, CIBERSORT and ssGSEA determined whether subtypes of tumor-infiltrating immune cells were associated with high or low risk.

### More sensitivity to chemotherapy for C3 subtype

GDSC (Genomics of Drug Sensitivity in Cancer) database was used to analyze chemotherapy response of HCC and various subtypes (Yang et al., 2013). We selected sorafenib, an approved chemotherapeutic drug, to predict the chemotherapy response to treat metastatic HCC. The prediction process was carried out by the R-packet “Prophet”, and the half maximal inhibitory concentration (IC50) was acquired by ridge regression and validation based on the GDSC training database (Geeleher et al., 2014).

### Statistical analysis

Kaplan-Meier curves and log-order tests were used to calculate survival in this study using R software (version 4.1.0). The *t*-test and Wilcoxon test of nonpaired students analyzed habitually and unusually distributed variables. Prognostic features, as well as other clinicopathological features, were assessed using ROC and C-index. A statistically significant difference was defined as one that is less than 0.05(*p* < 0.05).

## Results

### Identification of significant enriched lactate-related lncRNAs

**FIGURE 1:**
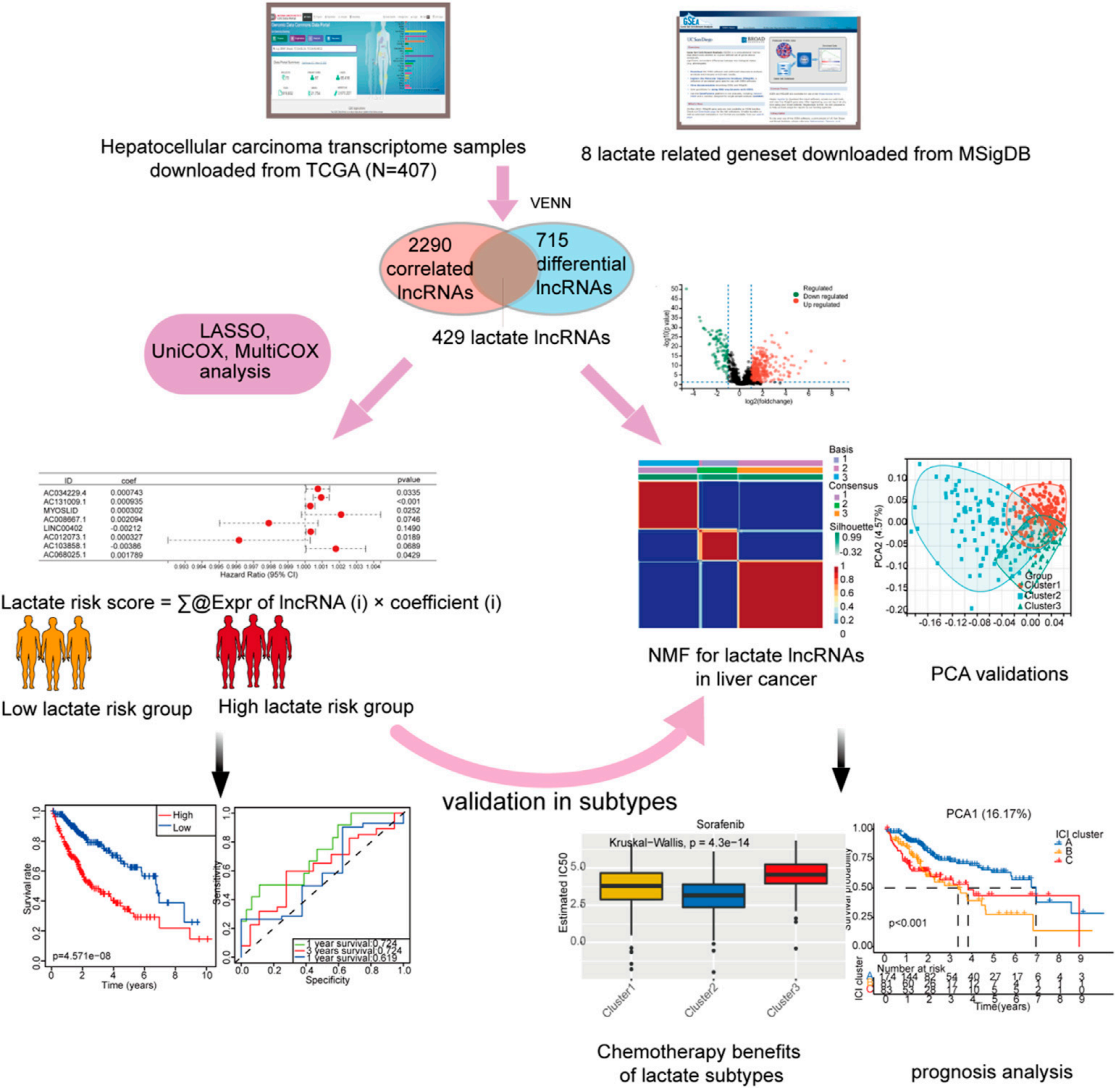
Overall flowchart of this study.

In this study, we systematically analyzed critical roles and predictive values of LM_lncRNAs in HCC using several advanced computational methods. The flow chart describing this work is shown in Figure 1.

MSigDB had eight GSEA datasets associated with lactate metabolism, and four were significantly enriched in HCC tissues:

HP_INCREASED_SERUM_LACTATE, HP_INCREASED_LACTATE_DEHYDROGENASE_LEVEL, HP_INCREASED_CSF_LACTATE, and HPABNORMAL_LACTATE_ DEHYDROGENASE_LEVEL (Supplementary Figurs S1).

**FIGURE 2:**
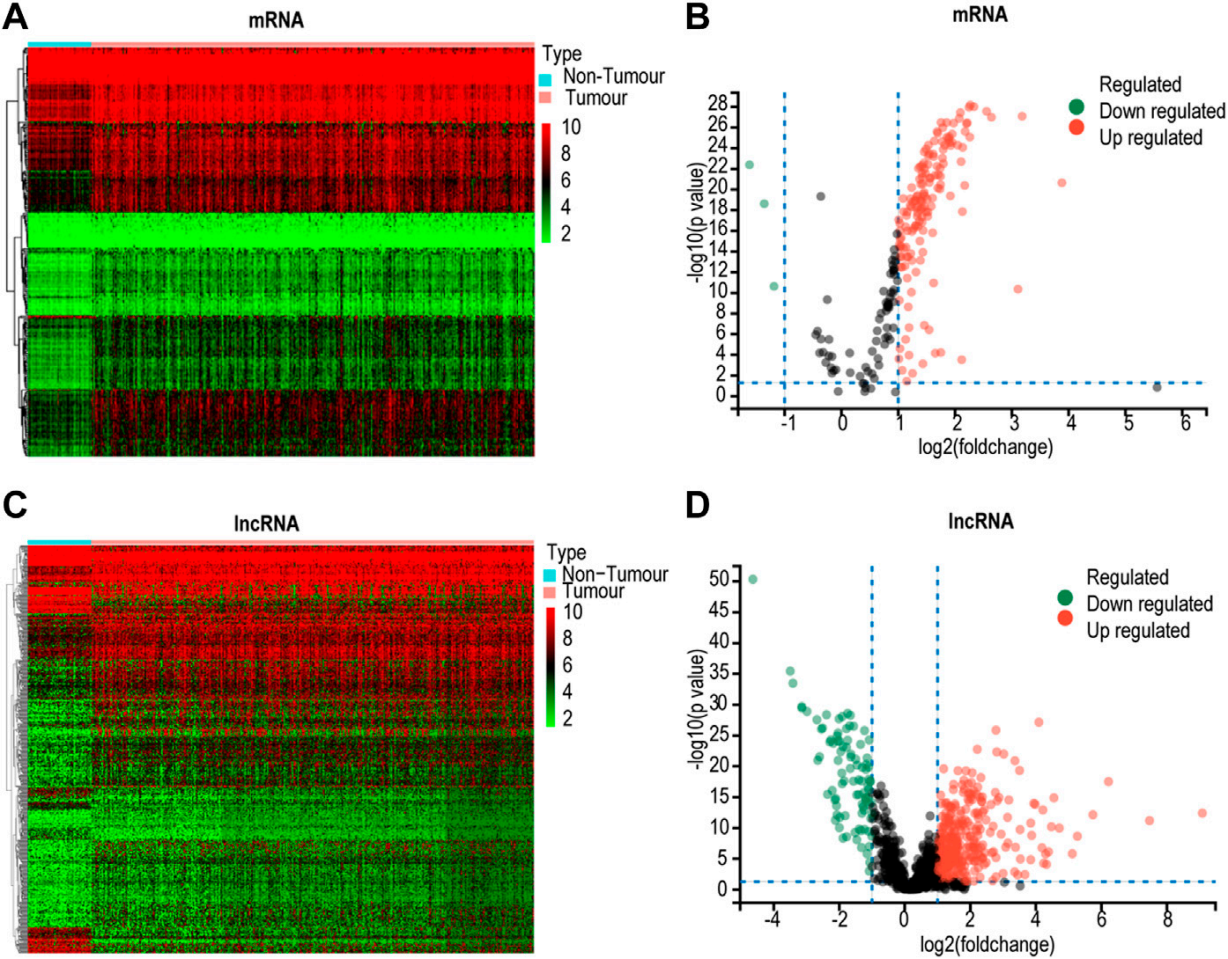
Screening for differentially expressed lactate genes. **(A)** Heatmap for differential lactate mRNAs. **(B)** Volcano plot for differentially expressed lactate mRNAs. **(C)** Heatmap analyses for differential lactate lncRNAs. **(D)** Volcano plot for differentially expressed lactate lncRNAs.

The database included 228 lactate mRNAs with significant enrichment pathways. After correlation analysis, 715 lactate pathway-enriched lncRNAs were retained after the intersection with the whole expression dataset of the sample. The screening criteria were Pearson coefficient >0.3 and *p* < 0.01. The expression of lactate mRNAs in HCC tissues and adjacent control tissues differed by 162, of which 159 were upregulated while three were downregulated (Figures 2A,B). In HCC tissues and adjacent control tissues, 429 lactate lncRNAs were differentially expressed, with 355 upregulated and 94 downregulated (Figures 2C,D). GO and KEGG analyses of lactic acid mRNAs in HCC are as follows.

The biological process category was enriched for mitochondrial respiratory chain complex I assembly, oxidative energy generation of organic matter, aerobic respiration, mitochondrial respiratory chain and metabolism of precursor metabolites, and NADH dehydrogenase complex. The cellular component was mainly enriched for concentrates on the mitochondrial respiratory chain, redox enzyme complex, and ADH dehydrogenase complex. The molecular function was mainly enriched for the NADH oxidase complex, REDOX enzyme complex, and active transmembrane transporter activity. KEGG analysis indicated that the upregulated genes primarily focused on thermogenesis, oxidative phosphorylation, reverse neural signaling, aminoacyl-tRNA biosynthesis, myocardial contraction, carbon metabolism, cofactor biosynthesis, tricarboxylic acid cycle, butyric acid metabolism, dicarboxylic acid metabolism, propionic acid metabolism, fatty acid degradation pathways, etc (Supplementary Figure S2).

### Identification of HCC molecular subtypes

**FIGURE 3:**
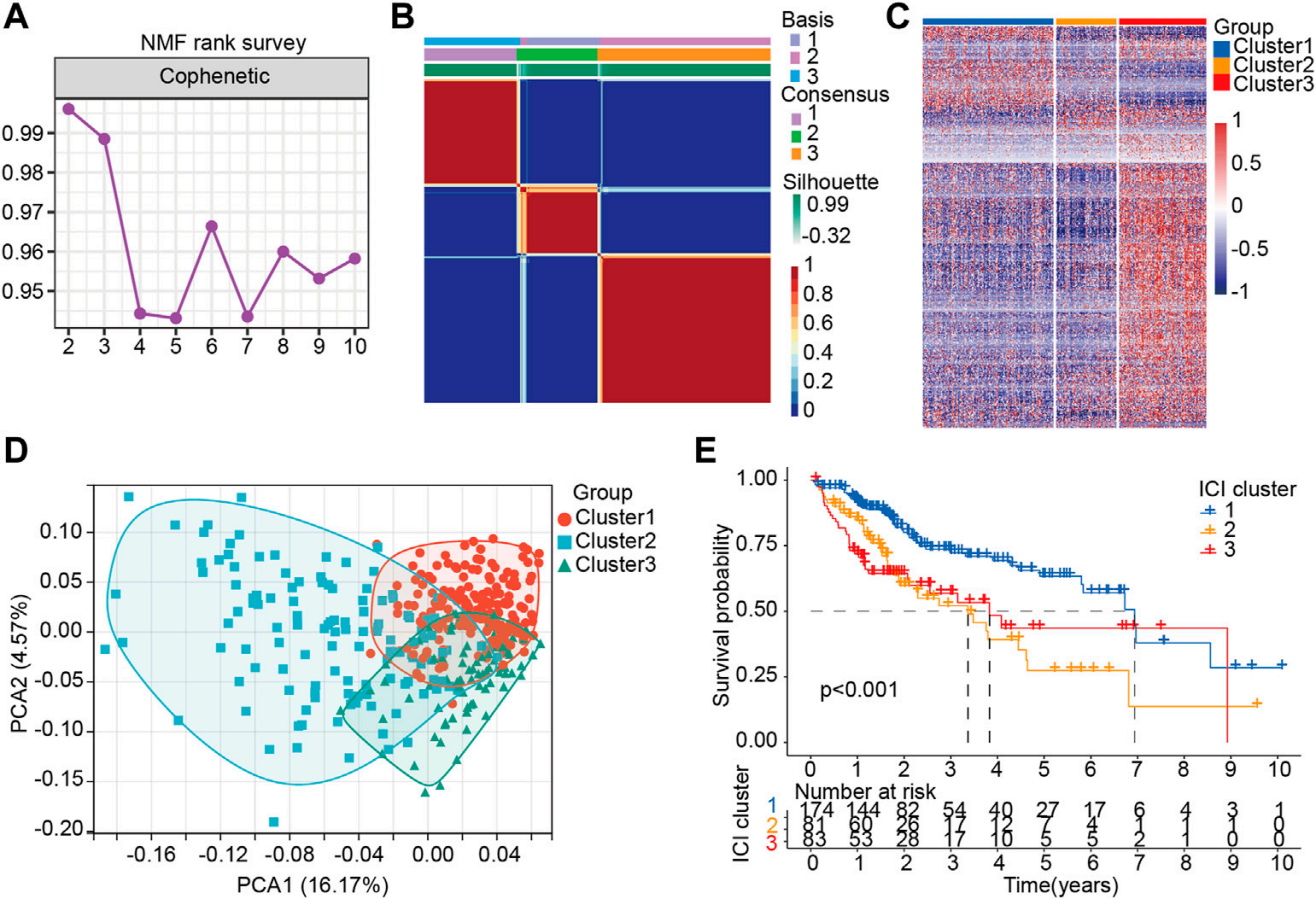
Stratification of HCC patients based on the lactate lncRNA. **(A)** NMF clustering using 426 lactate lncRNAs (k = 2–10). **(B)** The consensus map for k = 3. **(C)** The difference of LM-lncRNA expression in three groups was analyzed by Heatmap. **(D)** Principal components analysis (PCA) for patients based on lactate lncRNA expression. Each dot represents a patient, and different colors represent different immune subtypes. **(E)** Kaplan–Meier curves analysis for the three subtypes.

In order to determine the relationship between potential molecular subtypes and the prognosis of HCC, we examined the following. First, according to the expression characteristics of LM_lncRNAs, 429 lncRNAs with statistically significant differences (*p* < 0.05, |log_2_FC| > 1) were constructed into a matrix. Second, 407 samples from the TCGA cohort were included in all lncRNAs for the NMF common set. In order to determine k, the affinity coefficient was calculated, and the value of three was selected (the previous point with the most significant decline of the curve, cluster 1, cluster 2, and cluster three are listed C1, C2, and C3, respectively, Figures 3A,B). In Figure 3C, LM_lncRNA expression levels differ among C1, C2, and C3 subtypes. Most of the lncRNA was highly expressed in the C3 subtype and lowly expressed in the C2 subtype. Moreover, each subtype’s boundary is evident, meaning the subtype is robust and reliable. PCA was performed to distinguish the three subtypes, and samples were well separated by this PCA method (Figure 3D). We further evaluated the association between three subtypes and prognosis. A significant difference between three subtypes was found in the analysis. There was a significant difference in the overall survival (OS) between subtypes C1 and C2 (*p* < 0.001). However, the OS of subtype C3 was better after 4 years, a bias of the small sample size (Figure 3E).

**FIGURE 4:**
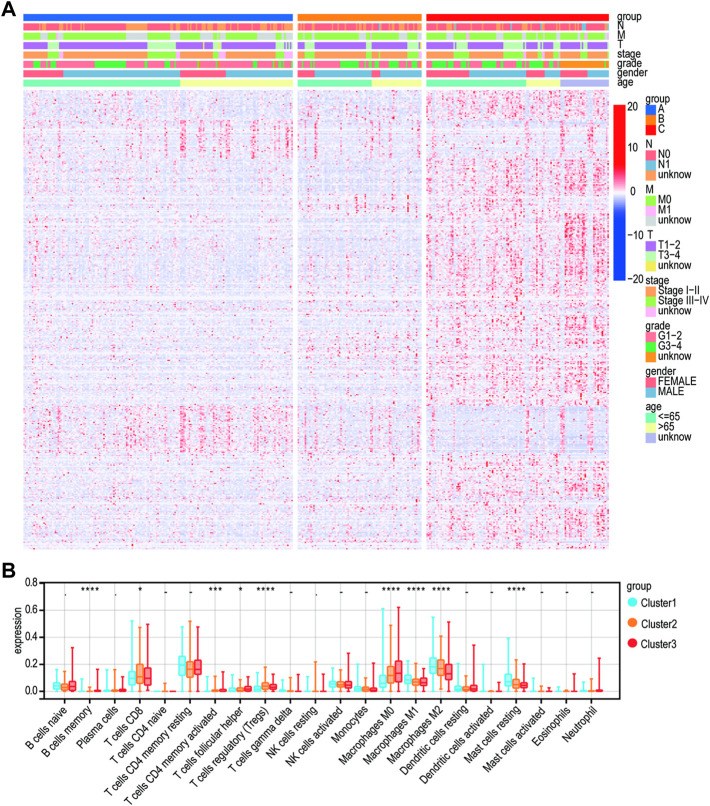
Clinical characteristics of the three subtypes. **(A)** Heatmap for the lactate lncRNA prognostic signature and clinicalpathological manifestations. **(B)** CIBERSORT for three subtypes.

**FIGURE 5:**
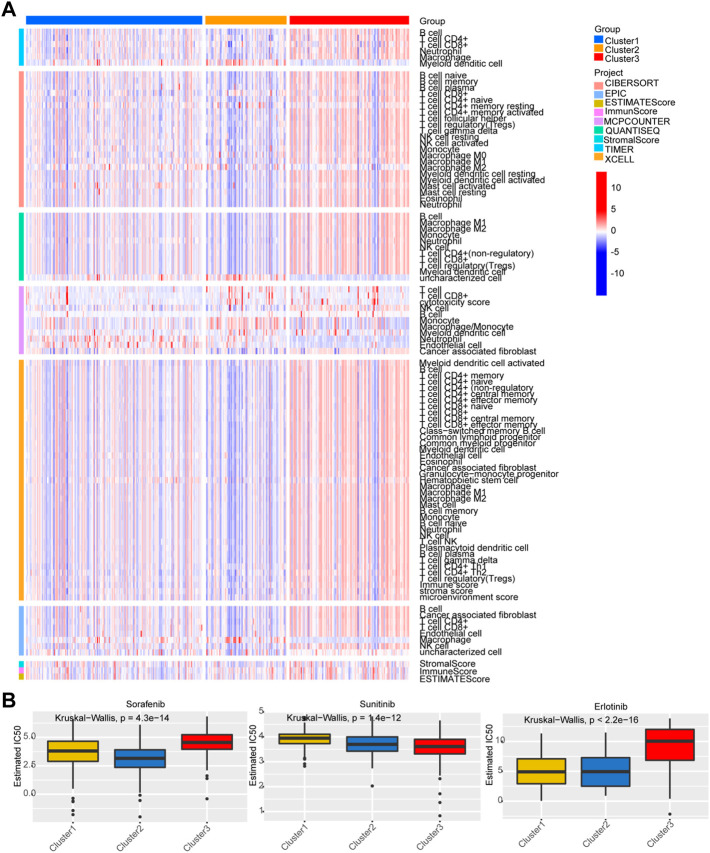
Analysis of immune status and chemosensitivity among the three subgroups. **(A)** Analysis of immune status. **(B)** Chemosensitivity analysis of three common chemotherapy drugs.

A heatmap was developed to describe the relationship between the expression of Lnc RNAs, molecular subtypes, and clinical factors in HCC. The proportion of stage III-IV and grade III-IV is 20.1 and 25.9% in the C1 group, respectively, both less than the proportion of C2 and C3. On the other hand, the proportion of T3-T4 in the C1 group is less than that in C2 and C3 groups. Notably, the C3 subtype is related to more patients with advanced stage disease and is prone to relapse or metastases, suggesting that patients with the HCC C3 subtype progressed rapidly (Figure 4A). Then, immune cell penetration levels, including CD8 T cells, B memory cells, and γδ T cells, were higher in the C3 subtype, whereas CD4 memory cells and M1 and M2 macrophages were significantly enriched in C1 (Figure 4B, Figure 5A). This is consistent with the notion that immune response suppresses cancer.

### Predicting chemotherapeutic response

The responses of the three subtypes to three chemotherapeutic drugs were assessed according to the GDSC database. The figure indicates that the C2 subtype was the most sensitive to the sorafenib, and the sensitivity of C3 subtypes to sunitinib was higher than that of the C1/C2. Compared with C3, C1 and C2 were more sensitive to erlotinib (Figure 5B).

### Construction and multivariate examination of the lactate-based lncRNAs prognostic signature

For the purpose of determining prognostic risk models, 374 HCC patients were included in the TCGA-HCC cohort and 249 LM_lncRNA sequences were analyzed. Using univariate Cox regression analysis, 63 OS-related genes for lactic acid metabolism were identified (*p* < 0.05). The Lasso regression analysis was then used to remove LM_lncRNAs that may be highly related to other LM_lncRNAs. Furthermore, 16 lncRNAs were modeled using the minimized *λ* method of Lasso Cox analysis (Supplementary Figures S3A, B). Furthermore, a prognostic signature model was constructed based on multivariate Cox regression analysis. Finally, eight lncRNAs were confirmed and applied to establish the lactic acid metabolism-related signature (Supplementary Figure S3C).

The predictive risk score formula evaluated the prognosis of each patient composed of eight LM_lncRNAs as follows:

Risk score = (0.0007423×Expr of AC034229.4) + (0.000935×Expr of AC131009.1) +(0.000302×Expr of MYOSLID) + (0.00209×Expr of AC008667.1) +(-0.00212×Expr of LINC00402) + (0.000327×Expr of AC012073.1) + (-0.00386×Expr of AC103858.1) + (0.00179×Expr of AC068025.1).

Six of these lncRNAs, including AC034229.4, AC131009.1, MYOSLID, AC008667.1, AC012073.1, and AC068025.1, all showed positive coefficients in Cox regression analysis, indicating that these six lncRNAs have high-risk characteristics because their high expression means that the patient has a shorter OS. The coefficients of LINC00402 and AC103858.1 were negative, indicating that these lncRNAs were protective.

**FIGURE 6:**
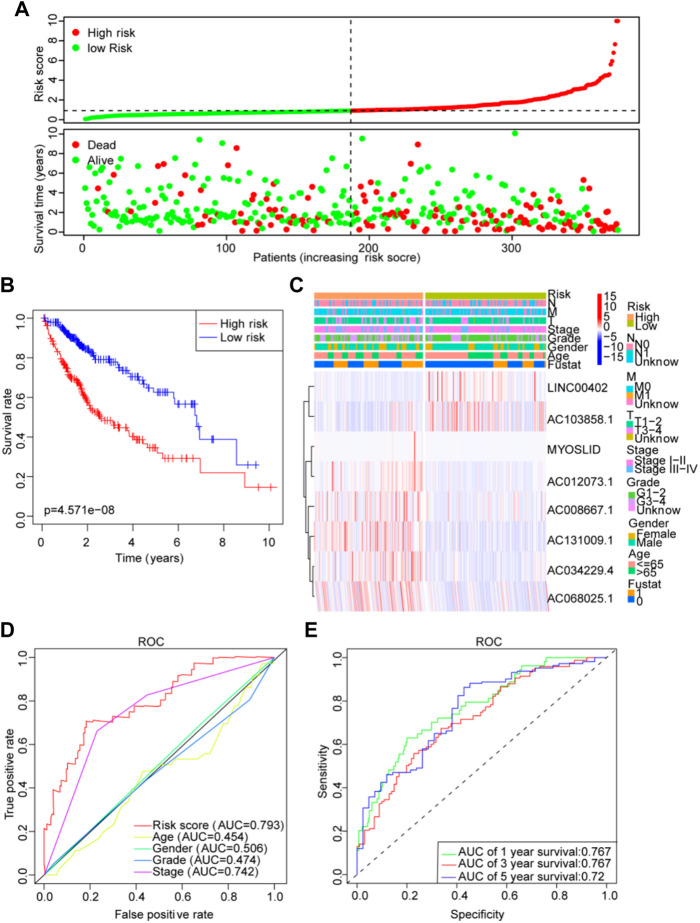
Lactate lncRNA signature based on TCGA. **(A)** Risk score distribution and survival status of the two risk groups. **(B)** Kaplan-Meier curve analysis for the cohort. **(C)** Heatmap for the lactate lncRNA prognostic signature and clinicopathological manifestations. **(D)** Timedependent receiver operating characteristic (ROC) analysis for 1-year overall survival (OS) based on eight lactate lncRNAs were compared to age, sex, grade, TNM stage, T stage, N stage, and M stage. **(E)** ROC analysis of the lactate prognostic signature at the 1-, 3-, and 5-year nomograms.

Then, patients’ risk scores were calculated according to this prognostic model. Based on the critical value of the median risk score, patients were divided into high-risk (n = 187) and low-risk (n = 187) groups for the LM_lncRNAs prognostic model. The risk score and survival status of these prognostic LM_lncRNAs are presented in Figure 6A. Kaplan-Meier test showed that the mortality rate of the high-risk rating group was relatively high. The survival time was relatively short (Figure 6B). A heatmap was used to analyze the connection between prognostic features of LM_lncRNAs and clinical pathology manifestations (Figure 6C). We could observe that the high expression of AC034229.4, AC131009.1, MYOSLID, AC008667.1, AC012073.1, and AC068025.1 was concentrated in clinical features with poor prognosis. In contrast, the LINC00402 and AC103858.1 were concentrated in clinical features with good prognoses. In addition, the area under the curve (AUC) of this characteristic lncRNA was 0.793, the most reliable indicator in the survival prediction of the model compared with age (AUC = 0.454), gender (AUC = 0.506), grade (AUC = 0.474), or stage (AUC = 0.742) (Figure 6D). Based on the AUC values of the novel lncRNAs, 1-, 3-, and 5-year survival rates were predicted to be 0.767, 0.767, and 0.72, respectively (Figure 6E). In addition, the three different subtypes were used for the validation of the lactic acid metabolism-related signature. Survival rates based on AUC for C1, C2, and C3 groups were 0.756, 0.756, 0.722; 0.724, 0.724, 0.619; 0.716, 0.716, 0.773, respectively (Supplementary Figure S4A). The high-risk OS rates of C1 and C3 were significantly higher than those of the low-risk rating group for these three subtypes (*p* < 0.05). Consistent with the results derived from the TCGA HCC database. Because of the small sample size, the C2 survival analysis results were not significantly different (Supplementary Figure S4B), indicating that the model has good stability.

### TCGA cohort nomogram construction and validation

In HCC patients, risk assessment could be an independent prognostic factor, according to single-factor and multifactor regression analyses (Supplementary Figure S5A, B). The nomogram based on the eight lncRNAs was shown in Supplementary Figure S5C. To predict the prognosis of patients with HCC, an effective prognostic nomogram was developed combining clinicopathological features with LM_lncRNA prognostic factors (Supplementary Figure S5D). 5-year and 3-year calibration curves (Supplementary Figure S5E) showed stable and accurate application in the clinical treatment of HCC patients.

To predict the potential functions of the eight LM_lncRNAs, we calculated the correlation value of lncRNAs and differentially expressed lactate-related mRNAs based on Pearson-related calculations. Moreover, |R2| > 0.3 and *p* < 0.001 were used as relevant criteria to construct a coding-noncoding gene coexpression network consisting of eight LM_lncRNAs and 48 mRNAs (Supplementary Figure S6A). The various pathways of high-risk and low-risk groups were analyzed using GSEA (Supplementary Figure S6B). Therefore, the low-risk group had the most prognostic LM_lncRNAs that can regulate retinol, tryptophan, fatty acid metabolism, and other metabolic pathways. Patients with high-risk characteristics are enriched in cell cycles, spots, and meiosis, as well as steroid hormone biosynthesis and PPAR signaling.

### Genomic profiling and immune infiltration levels by subtypes

**FIGURE 7:**
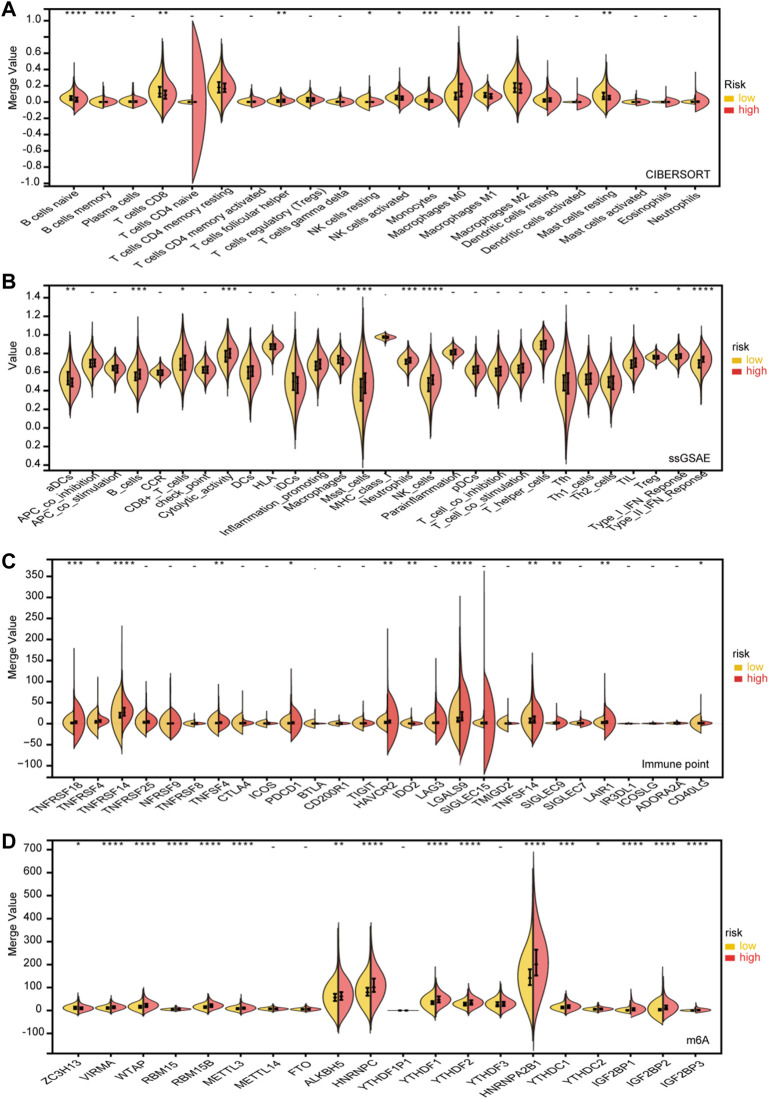
Immune infifiltration level analysis for the high- and lowrisk groups. **(A)** CIBERSORT. **(B)** ssGSEA. **(C)** Expression of immune checkpoints between the high- and low-risk subgroups. **(D)** Expression of m6A genes between high- and low-risk subgroups (*p* < 0.1, *p < 0.05, **p < 0.01, ***p < 0.001, ****p < 0.0001).

An immune infiltration analysis is conducted using a risk model that includes NK cells, B cells, and related functions in order to determine the relationship between immunity and the model. In CIBORSORT, M0, M1 macrophages, T cells, and B cells showed significant differences. Furthermore, the low-risk and high-risk groups showed significant differences in T-cell function and type II IFN response based on single-sample gene collection and enrichment analysis (ssGSEA) (Figures 7A,B). After evaluating the differences in immune checkpoint expression between the two groups, we found significant differences in the gene expression of VIRMA, WTAP, RBM15, RBM15B, YTHDF1, YTHDF2, and HNRNPA2B1 between the two groups (Figure 7C). The expression of m6A-related mRNA differed significantly between the relatively high-risk and low-risk groups, including ALKBH5, HNRNP, HNRNPA2B1, YTHDC, IGF2BP1, IGF2BP2, and IGF2BP3 (Figure 7D).

## Discussion

In addition to genetic and epigenetic changes in regulatory genes, HCC is a multistep and complex process. The most common clinical tumor markers used to diagnose HCC are alpha-fetoprotein (AFP) and carcinoembryonic antigen (CEA). Both of these markers are not particularly specific (Ching et al., 2015), so researchers are now focusing on finding a more specific HCC diagnostic marker. Identifying cancer-associated molecules may lead to discovering novel therapeutic targets and biomarkers (Shimizu et al., 2017). Considering the complex pathophysiology of HCC, it is not likely that a single ideal biomarker can be identified. An ideal approach would be to identify the specific phenotype associated with a biomarker and its underlying mechanism. A combination biomarker, consisting of several markers, has additionally been demonstrated to improve prediction accuracy. Despite aerobic conditions, malignant tumor cells are known to consume more glucose and produce more lactic acid, which results in an accumulation of lactate in the HCC microenvironment. Previous studies have focused on glycolysis-driven genes in tumor development, inflammation, and immune regulation (Yuan et al., 2014; Pucino et al., 2019; Zheng et al., 2020). The prognostic potential of LM_lncRNAs in HCC has not been thoroughly investigated (Goetze et al., 2011).

According to our findings, single lncRNA was significant but not strong enough to undertake personalized diagnosis, while LM_lncRNAs combination markers improved the diagnostic accuracy.

In this work, we constructed a novel high-efficiency eight LM_lncRNA signature based on the TCGA dataset to evaluate HCC lactate subtypes. The results of ROC analysis on the TCGA dataset confirmed that our model has a high prognostic value. Because the risk signature relies on the expression levels of eight LM_lncRNAs, it is more economically and clinically feasible than whole-genome sequencing. Based on LM_lncRNAs, the NMF algorithm was used to perform consistent clustering of HCC patients. The three molecular subtypes (C1, C2, and C3) have already passed TCGA rank evaluation and verification. Therefore, depending on the molecular subtype, there is a noticeable difference in OS and immune levels. The Kaplan–Meier curve results showed that in patients with advanced-stage disease, subtype C1 was associated with prolonged survival, while subtype C3 was associated with shortened survival. However, C3 samples were more sensitive to sunitinib chemotherapy. In addition, the model is significantly correlated with clinical factors, which further supports the reliability of its prognostic value. Studies have shown that LM_lncRNAs can be used as independent predictors for OS. The nomogram constructed from staging, grading, and signature shows good predictability over 1, 3, and 5 years, which may help facilitate individualized treatment of HCC patients. The application of nomograms that integrate gene signatures with clinicopathological parameters can help clinicians evaluate the prognosis of individual patients with greater accuracy. As a result, C-index, ROC curve, and standard curve indicate that the risk model constructed in this study has a high level of robustness and reliability. Moreover, the construction of the lactic acid model can also help the clinical design of drugs with lactic acid lncRNA as a target and improve the survival rate of patients.

Lactate metabolism has been associated with the immune microenvironment further to explore the relationship between the TME and subtypes. A comparison of the infiltration of 24 immune cell types (including 18 T-cell subtypes) in C1, C2, and C3 with six other immune cell types showed that macrophage levels of M0, M1, and M2 in C2 and C3 were significantly lower than in C1. Clinical and experimental evidence suggests that tumor-associated macrophages (TAMs) are central to immunosuppressive cells and cytokine networks and are associated with the development and metastasis of tumors and immune escape (Pollard, 2004). TAMs have multiple functions during tumorigenesis depending on their activation state, polarizing from an M1 phenotype to an M2 phenotype. The M2 phenotype is mainly used for tissue repair and remodeling, immunomodulation, and tumor-promoting roles (Mills et al., 2000). Through innate signals such as TGF-β and IL-10, T cells are induced to switch to Treg without anticancer activity (Mantovani and Sica, 2010). This may partly explain why C2 and C3 survived less than C1. Our findings are very similar to recent findings that included hypoxia and immune genes. OS was significantly better in the high hypoxic/immune-low group (*p* < 0.01) than that in the hypoxic/immune-high group (*p* < 0.01) (Zheng et al., 2020).

Among the eight lncRNA signatures, MYOSLID, AC012073.1, and LINC00402 were associated with progression in various cancer types, but there is hardly any information reported on HCC. MYOSLID was identified as a lncRNA that promotes invasion and metastasis by modulating a partial epithelial-interstitial transformation procedure for head and neck squamous cell carcinoma (Xiong et al., 2019). Furthermore, this lncRNA is associated with autophagy genes and is critical for osteosarcoma progression and the occurrence and development of gastric cancer (Han et al., 2019). In addition, autophagy-related lncRNAs are known to be prognostic indicators of head and neck squamous cell carcinoma (Zhou et al., 2021). Based on the above reports, this lncRNA is associated with cancer initiation and spread, can predict cancer prognosis reasonably and may become a potential molecular target. The results show that AC012073.1 has a critical prognostic value in esophageal squamous cell carcinoma (Zhang et al., 2021). LINC00402 and other lncRNAs enhance PHLPP2 expression by competing with the endogenous RNA network and exerting repression in colon cancer pathogenesis (Wu et al., 2020). A similar result has shown that this lncRNA is involved in the regulation of ceRNAs in metastatic melanoma and affects the prognosis of patients (Wang et al., 2019).

The N6-methyladenosine (m6A) modification mechanism is closely related to tumorigenesis, protein translation, and drug response (Deng et al., 2018). For future translational research in cancer therapy, this work also explored the relationship between aberrant expression of the m6A regulator mRNA profile and prognostic features of HCC and its potential role in cancer therapy. The m6A methyltransferase complex comprises METTL13, METTL14, and WTAP. It may also include VIRMA and RBM15, acting as m6A writers, demethylases acting as erasers, and m6A-binding protein (YTHDF1/2/3, METTL3) readers, which determine the fate of the target mRNA transcription of m6A modifications (Zhang et al., 2020b). This study includes VIRMA, WTAP, RBM15, RBM15B, ALKBH5, HNRNPC, METTL3, YTHDF1, YTHDF2, HNRNPA2B1, YTHDC1, IGF2BP1, IGF2BP2, and IGF2BP3 were upregulated in the high-risk group. The expression of METTL3 and METTL14 in m6A writers and the development of HCC have been examined in recent studies (Ma et al., 2017; Chen et al., 2018). Some functions of METTL3 were not related to m6A because of the opposite effect of METTL3 and METTL14 on HCC cell migration. A high level of METTL3 indicates a poor prognosis, while the level of METTL14 mRNA shows an opposite trend, which is in accord with a previous report (Ma et al., 2017). According to a previous study, downregulation of 14 is a poor indicator of recurrence-free survival in HCC, and its expression level correlates closely with tumor spread. By regulating the microprocessor protein DGCR8, METTL14 promotes the maturation of PRI-mir126 into mature mir126, a tumor suppressor of HCC metastasis (Chen et al., 2018).

More recently, lncRNAs have also been shown to have essential roles in tumor-intrinsic mechanisms for immune suppression (Tang et al., 2019). Future studies must explore the function of these LM_lncRNAs in tumor immunity and as predictive immune checkpoint inhibitors for immunotherapy.

## Conclusion

A novel and efficient LM_lncRNAs signature and model were established, confirmed to have high prognostic value by ROC analysis, and may be used as independent predictors of OS.

Finally, some critical LM_lncRNAs were significantly clustered in oxidative respiration pathways, such as aerobic respiration and NADH oxidation, which should facilitate the ongoing effort to understand the role of lncRNAs in the lactate metabolism immune response. The present findings may provide a rationale for designing novel lactate metabolism-related targeted therapies by optimizing promising therapeutic markers and targets.

## Data Availability

The datasets presented in this study can be found in online repositories. The names of the repository/repositories and accession number(s) can be found in the article/Supplementary Material.
