# Supplementary material for: Identification of novel lactate metabolism signatures and molecular subtypes for prognosis in hepatocellular carcinoma
Source: Front Cell Dev Biol. 2022 Sep 2;10:960277. doi: 10.3389/fcell.2022.960277 (PMC9486814; doi:10.3389/fcell.2022.960277)
Supplement: Supplementary file 1 [file DataSheet1.pdf]

Supplementary Material

1 Supplementary Figures

Supplementary Material

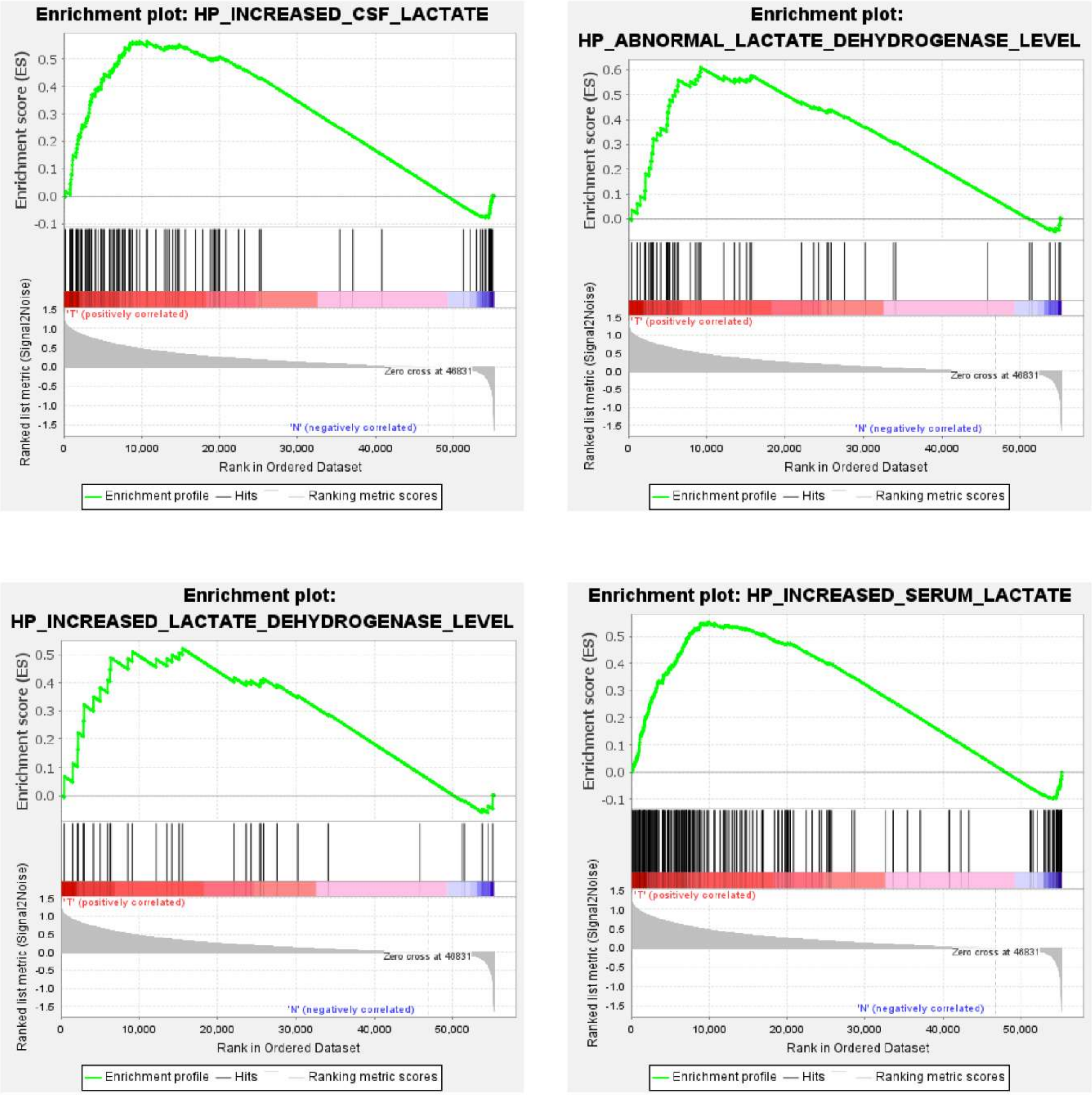

Supplementary Figure S1 Differentially expressed pathway mRNAs between hepatocellular carcinoma (HCC) and paracancerous tissues.

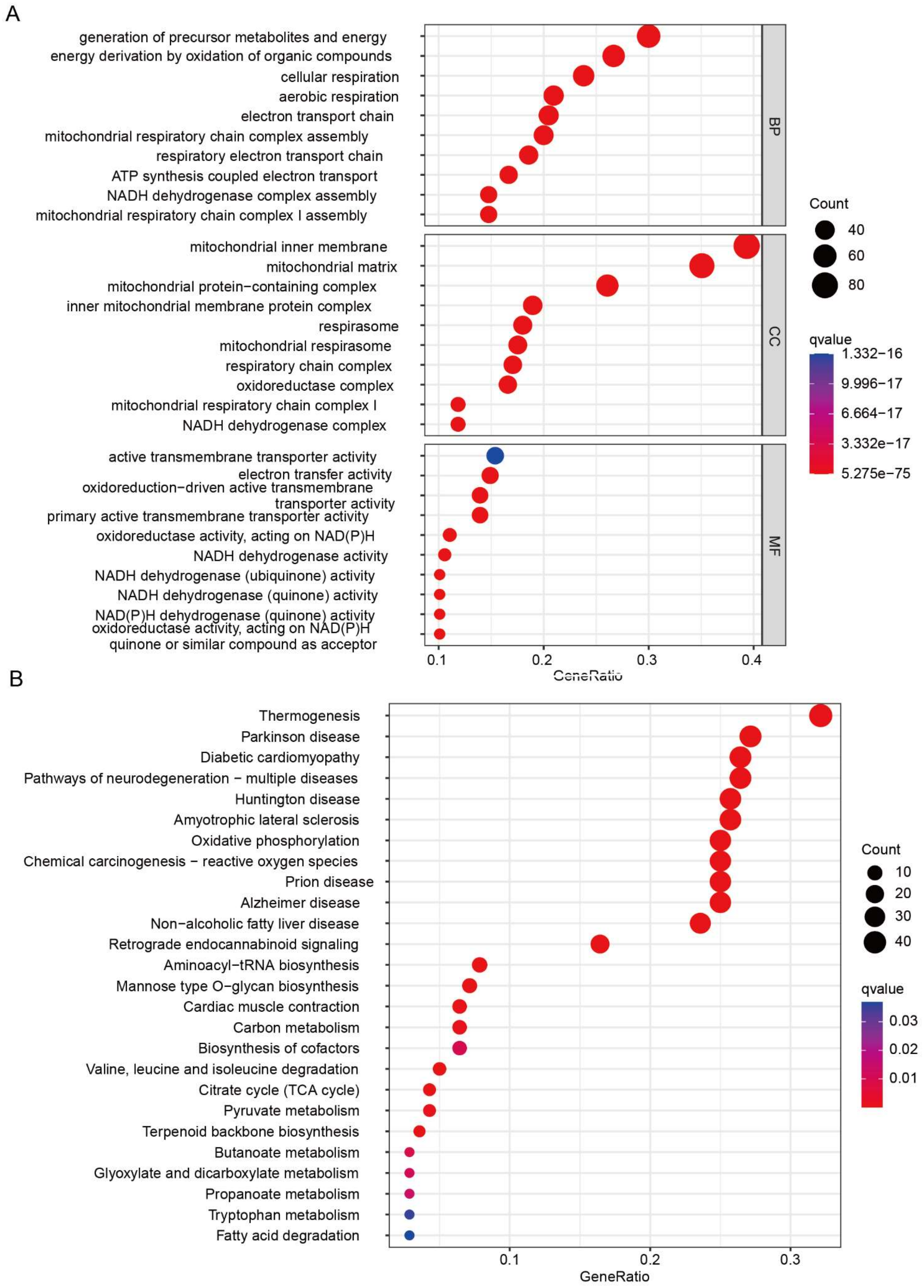

Supplementary Figure S2 Functional enrichment analysis between high- and low-risk subgroups. **(A)** Gene ontology (GO). **(B)** Kyoto Encyclopedia of Genes and Genomes (KEGG) analyses of lactate mRNA in hepatocellular carcinoma.

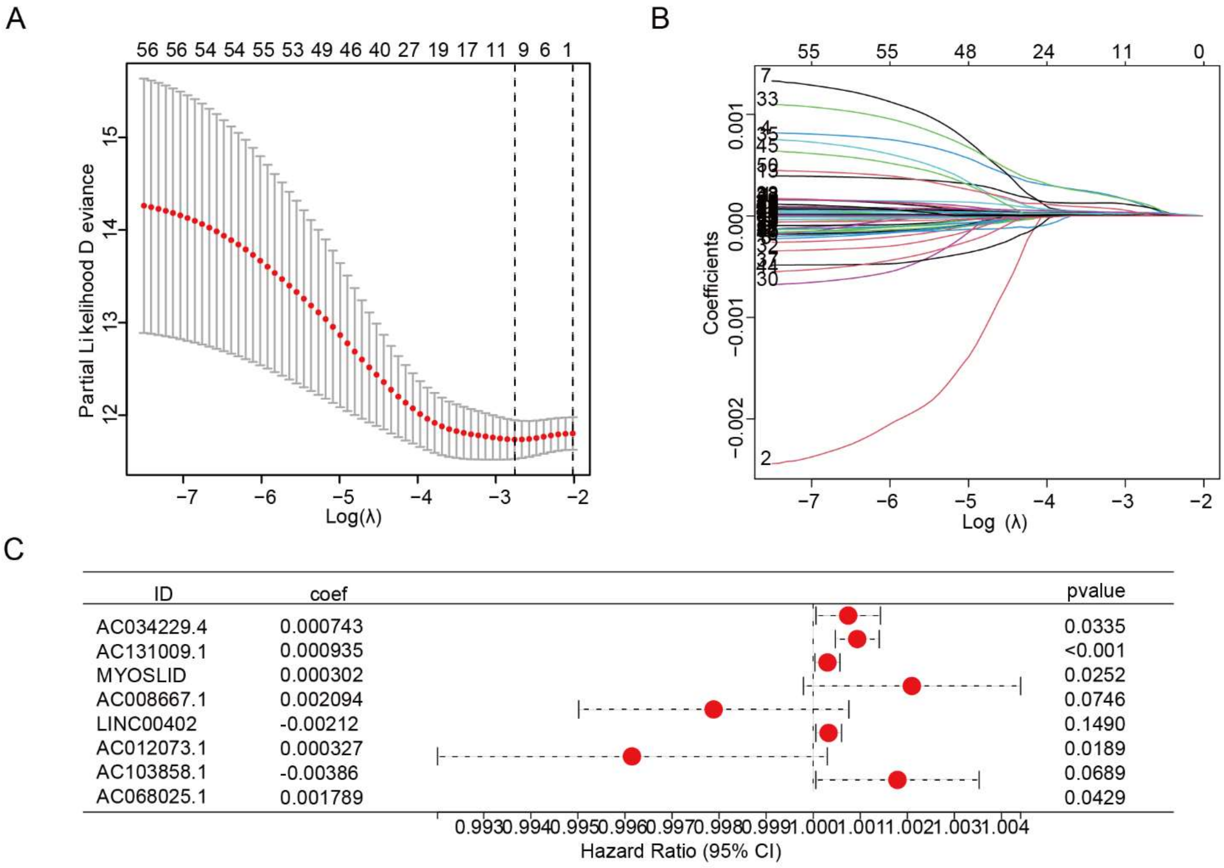

Supplementary Figure S3 Construction of a prognostic lactate lncRNA model. **(A)** Validation for Lasso regression. **(B)** Lasso coefficients of lactate lncRNA. **(C)** Multivariable Cox regression analysis of differentially expressed genes. **(D)** Forest plots for multivariate Cox analysis between lncRNA expression and OS.

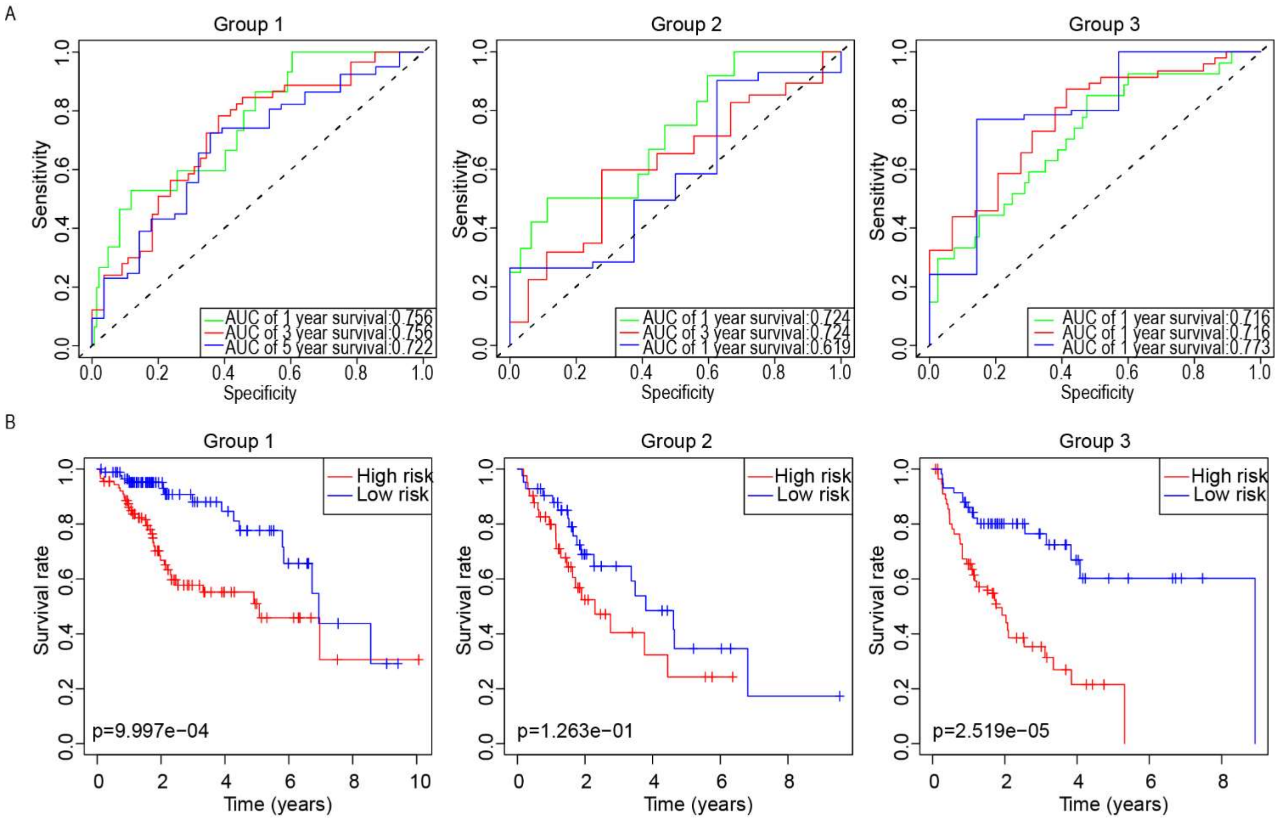

Supplementary Figure S4 Validation in the subtypes for the lactate lncRNA prognostic signature. **(A)** ROC analysis for the lactate lncRNA signature in the subtypes. **(B)** Kaplan-Meier curve analysis for the OS in the subtypes.

Supplementary Material

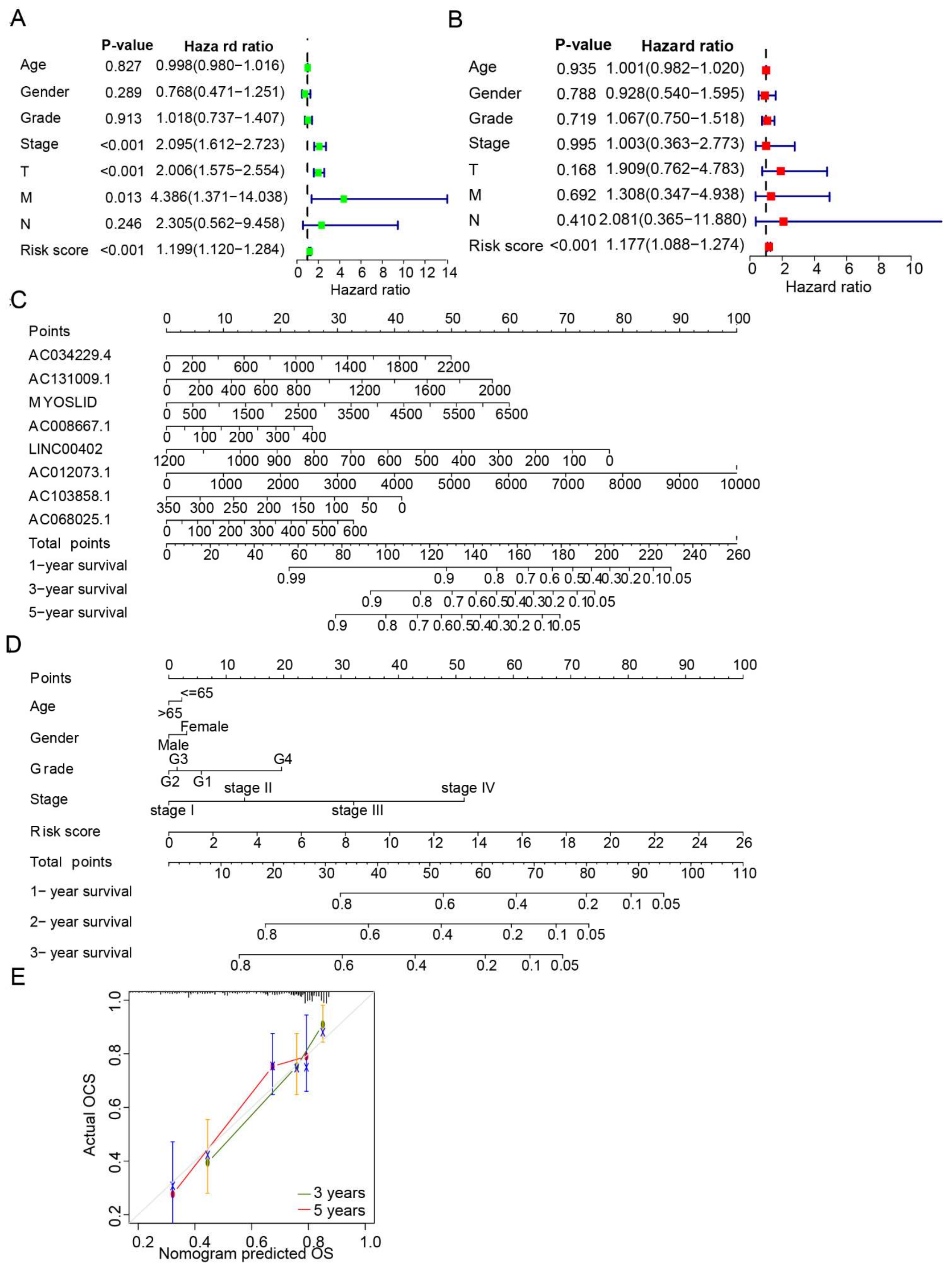

Supplementary Figure S5 Construction and validation of the forest plot and nomogram based on lactate lncRNAs. **(A)** Forest plots showing univariate Cox analysis between lncRNA expression and OS. **(B)** Forest plots showing multivariate Cox analysis between lncRNAs and OS. **(C)** A nomogram for the lactate lncRNA signature. **(D)** A nomogram for both prognostic lactate lncRNA and pathological factors. **(E)** C-index analysis of the nomogram.

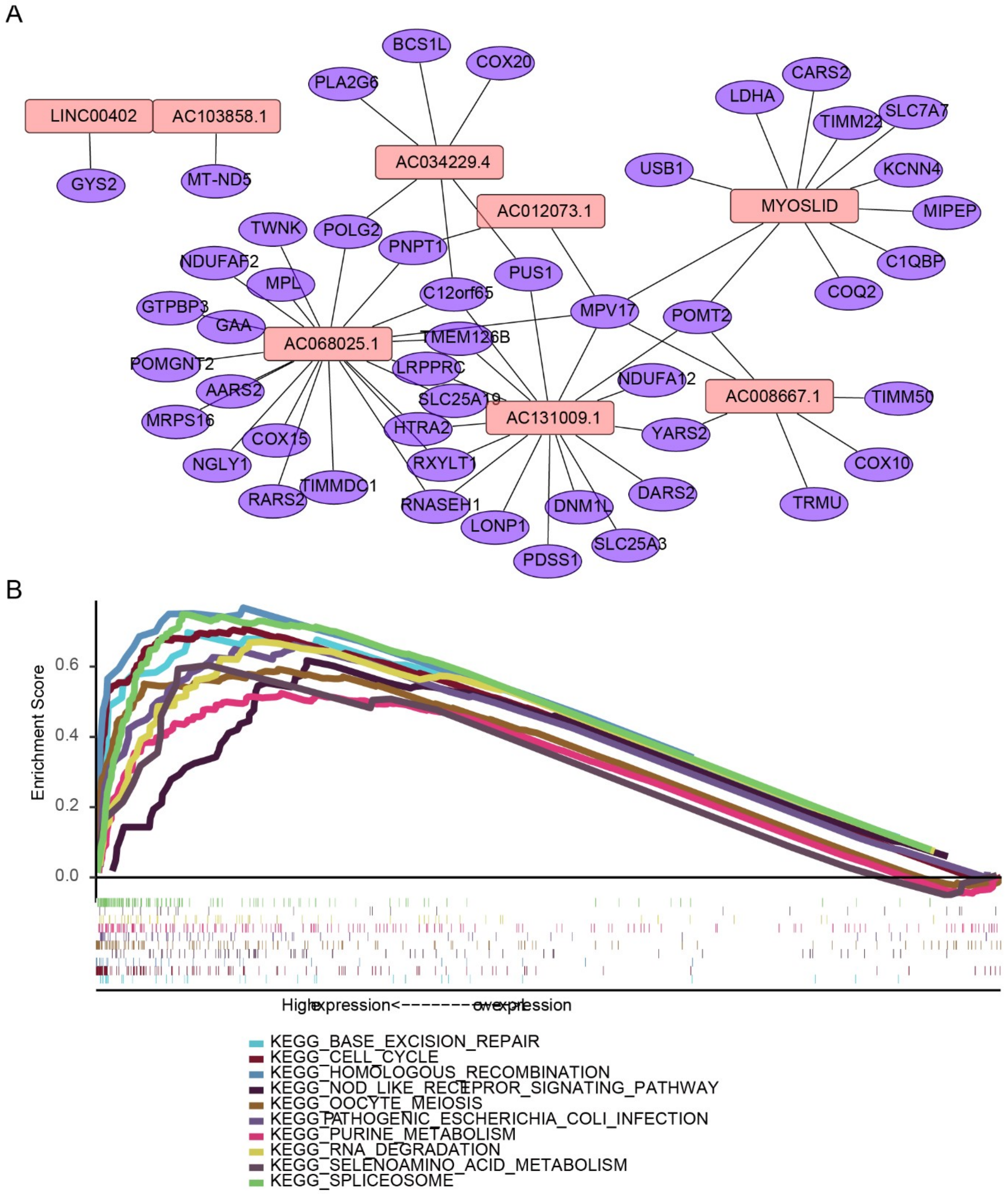

Supplementary Figure S6 Lactate lncRNA and mRNA function and network. **(A)** Protein–protein interaction networks between the identified lncRNA and mRNA expression. **(B)** Gene enrichment analysis for lactate mRNA based on TCGA.
